# Supplementary material for: A Pilot Study of Multiplex Ligation-Dependent Probe Amplification Evaluation of Copy Number Variations in Romanian Children with Congenital Heart Defects
Source: Genes (Basel). 2024 Feb 5;15(2):207. doi: 10.3390/genes15020207 (PMC10887610; doi:10.3390/genes15020207)
Supplement: Supplementary file 1 [file genes-15-00207-s001.zip › genes-2837477-supplementary.pdf]

Table S1. SALSA MLPA Probemix P311-B2 Congenital Heart Disease probe sequences

Gene	Exon	Partial sequence [1]	Length (nt)
GATA4	Upstream	CATGCTCAAGAT-AGGCACTGGAGC	362
	Upstream	GAGGTTCTTCTT-TAAAATCCATTC	229
	Exon 1	TTTCTTCCCTTT-CTTTGCTCCTTC	148
	Exon 3	CTCAGTAGATAT-GTTTGACGACTT	337
	Exon 4	CTACATGAAGCT-CCACGGGGTACG	355
	Exon 5	AAGAACCTGAAT-AAATCTAAGACA	142
	Exon 6	CAACTCCAGCAA-CGCCACCACCAG	238
	Exon 7	CACAAGGCTATG-CGTCTCCCGTCA	202
	Downstream (*CTCB* gene)	AAGTGTAGCAAG-ATCTGTGAGCCT	130
NKX2-5	Exon 1	CCGGCCAAGTGT-GCGTCTGCCTTT	274
	Exon 1	AGGTGAGGAGGA-AACACAGGCCCC	190
	Exon 2	CGCTCCAGCTCA-TAGACCTGCGCC	427
	Exon 2	CGGGATTCCGCA-GAGCAACTCGGG	266
TBX5	Exon 1	GACGTTGGAAGA-AGACCTGGCCTA	166
	Intron 1	CTATTCTGGGTA-AGCAGTAAACCC	317
	Exon 2	GCCTGACGCAAA-AGACCTGCCCTG	184
	Exon 3	AATCAAAGTGTT-TCTCCATGAAAG	195
	Exon 5	TCCTTCCAGAAA-CTCAAGCTCACC	346
	Exon 6	TACCAGCCTAGA-TTACACATCGTG	247
	Exon 8	GTGAGGCAAAAA-GTGGCCTCCAAC	160
	Exon 8	CCATTGTACCAA-GAGGAAAGGTGA	283
	Exon 9	AAGAAGATTCCT-TCTACCGCTCTA	174
	Exon 9	TTTGCTTTGGTT-TTGTCCTGCCTT	303
BMP4	Exon 1	TGCAGGGACCTA-TGGTGAGCAAGG	255
	Intron 1	CGCAGGCCGAAA-GCTGTTCACCGT	436
	Exon 3	TGGTAACCGAAT-GCTGATGGTCGT	454
	Exon 4	AACATCTGGAGA-ACATCCCAGGGA	391
CRELD1	Exon 4	CAAGTCAGACTT-CGAGTGCCACCG	136
	Exon 11	GCATTCCCCATC-TTAACTGATTTA	408
22q11.2 region	*CDC45* gene	ATGTTCGTGTCC-GATTTCCGCAAA	466
	GP1BB gene	CACAACCGAGCT-GGTGCTGACCGG	208
	DGCR8 gene	GACTCAGCGACT-GCACCAGTGGCA	382

nt, nucleotides;

[1] 24 nucelotides adjacent to ligation site
